# Beta-thalassaemia major – a public health problem in Malaysia: impacts, coping strategies and needs of parents with affected children

**DOI:** 10.1186/1471-2458-12-S2-A29

**Published:** 2012-11-27

**Authors:** Nursalihah Muhammad, Jin Ai Mary Anne Tan, Elizabeth George, Wong Li Ping

**Affiliations:** 1 Department of Molecular Medicine, Faculty of Medicine, University of Malaya, 50603 Kuala Lumpur, Malaysia; 2 Department of Pathology, Faculty of Medicine and Health Sciences, Universiti Putra Malaysia, 43400 Serdang, Malaysia; 3 Department of Social and Preventive Medicine, Faculty of Medicine, University of Malaya, 50603 Kuala Lumpur, Malaysia

## Background

Thalassaemia is a genetic blood disorder which can be life-threatening. About 4% of Malaysians are β-thalassaemia carriers. Malaysia has a multiracial population with diversity in religious and cultural beliefs. This study aims to identify challenges and solutions associated with the impacts, coping strategies and needs of parents with β-thalassaemia major children.

## Materials and methods

In-depth interviews were conducted with 37 multi-ethnic parents (21 Malays; 7 Chinese; 9 indigenous groups) consisting of 20 from urban and 17 from rural populations. Interviews were conducted in University Malaya Medical Centre and hospitals in Miri, Limbang and Lawas in Nothern Sarawak.

## Results

The respondents from both populations were equally knowledgeable about thalassaemia, management and prevention. The majority of parents accepted thalassaemia major as a genetic disorder from both partners. Although all parents recognise prenatal diagnosis as necessary for determination of foetal status and/or prevention, about two-third of parents were not receptive as they believed termination of pregnancies was against their religion. A few urban working parents encountered difficulties with employers due to frequent days-off while the majority managed with partners taking turns for hospital visits. About one-third of working urban mothers resigned to care for their child/children. This was not a problem with rural parents as the majority of mothers were housewives. All parents listed emotional stress as the dominant reaction when informed that their child/children were affected. Strategies employed to cope include acceptance of the disorder, seeking information and reliance on religious convictions. The majority of Sarawakian parents requested government financial aid as their average salary was below RM850.

## Conclusion

This study demonstrates that early dissemination of information on thalassaemia and treatment will assist both urban and rural parents to accept and cope with management of the disorder and also lessen their anxiety. Malaysian health systems should also provide financial assistance to parents in rural areas due to their low incomes.

